# How do aged-care staff feel about antimicrobial stewardship? A systematic review of staff attitudes in long-term residential aged-care

**DOI:** 10.1186/s13756-022-01128-5

**Published:** 2022-06-28

**Authors:** Saniya Singh, Chris Degeling, Dominic Fernandez, Amy Montgomery, Peter Caputi, Frank P. Deane

**Affiliations:** 1 School of Psychology, University of Wollongong, Wollongong, NSW Australia; 2 Australian Centre for Health Engagement, Evidence and Values, School of Health and Society, University of Wollongong, Early Start Building 21.110, Wollongong, NSW 2500 Australia; 3 School of Nursing, University of Wollongong, Wollongong, NSW Australia

**Keywords:** Antimicrobial resistance, Prescribing, Stewardship, Residential aged-care, Healthcare workers, Attitudes of health personnel, Education, Perception of risk, Risk feelings

## Abstract

**Background:**

Antimicrobial resistance (AMR) is a problem in residential aged care facilities (RACF). There is a gap in our understanding of how psychosocial barriers such as risk perceptions shape staff attitudes towards antimicrobial stewardship (AMS). We sought to ascertain the attitudinal domains that have been identified to be of importance to AMS in RACF and comment on how they have been measured empirically. Our aim was to consolidate what is known regarding staff attitudes and perceptions in order to inform future stewardship.

**Method:**

We searched PsycINFO, PsycARTICLES, CINAHL Plus, MEDLINE, PubMed, Web of Science, Cochrane, and Scopus databases for primary studies of healthcare workers attitudes to AMS in RACF (1990-February 2021).

**Results:**

14 Studies were included in the review, within which 10 domains were identified: attitudes towards antimicrobial prescribing; guidelines; educational interventions; self-confidence regarding clinical assessment and prescribing; awareness of AMR as a problem and stewardship as a priority; self-efficacy; perception of role; perception of risk; team culture and resident/family members expectations. 46 measures were developed across the 14 studies to measure the 10 domains. The variability in the attitudinal domains and how these domains were measured was large. Only 13% included psychometric data regarding reliability and/or internal consistency.

**Conclusions:**

Attitudes are generally defined as having three evaluative bases: cognitive, behavioural, and affective. Findings from the current review suggest that the measures commonly used to capture healthcare staff attitudes to AMS do not sufficiently capture affect; particularly with respect to staff’s risk perceptions, perceptions of their role, and family members’ expectations. Given that affective processes have been postulated to influence medical decision making, these findings highlight the importance of understanding how staff, especially nurses feel about implementing AMS strategies and other peoples’ (e.g. residents and their families) perceptions of stewardship. It is expected that a more nuanced understanding of RACF nurses affective experiences when applying AMS, and their perceptions of the risks entailed, will help in reducing barriers to overprescribing antibiotics.

**Supplementary Information:**

The online version contains supplementary material available at 10.1186/s13756-022-01128-5.

## Introduction

Interventions to improve antimicrobial stewardship (AMS) and address antimicrobial resistance (AMR) in residential aged-care facilities (RACF) have included [10]: the development and dissemination of local guidelines; the use of resident assessment/communication forms; use of antibiograms microbiological testing algorithms; educational interventions for prescribers (e.g. physicians and nurse practitioners) and residents and their relatives. A recent meta-analysis concluded that stewardship reduced antimicrobial use by 14% in RACF [64]. However, long-term sustainability and cost-efficiency have not been measured [10, 22, 23, 45]

Despite sustained efforts, antibiotic prescribing in RACF is highly variable and giving antibiotics to residents without assessment by a doctor remains a common-practice [20, 40, 41, 24]. RACF staff manage high levels of potential urinary tract, respiratory and wound infections in the residents. The diagnostic uncertainty in an older population places pressure on staff to balance the risk of unnecessary antibiotic use following misdiagnosis against the potential harms of not treating an infection in a timely manner [40]. How staff perceive and respond to risk has been identified as a significant barrier to reducing overprescribing in aged-care [6, 13]. Perceptions of healthcare-related risks in aged-care settings are often influenced by resident vulnerabilities (multiple comorbidities, compromised immune systems, cognitive decline), systemic challenges (access to prescribers and diagnostic testing) and pressure from residents (and their family members) [31, 47].

Nurses have been identified as central to GP decision-making about treatment in RACF contexts [15, 52]. Role responsibilities include instigating the escalation of care when needed (e.g. urine culturing), collaborating with and relaying information between prescribers and residents (and their families), and resident advocacy [14, 26]. A number of studies highlight nurses’ concerns regarding a lack of collaboration and resistance from prescribers as barriers to stewardship [12, 22, 29, 41]. Whereas, prescribers’ report a lack of confidence in nurses’ accounts of symptoms, and pressure from nursing staff in favour of prescribing [55, 66].

Against this background, diagnostic uncertainty has been recognised as the most common source of anxiety for physicians across specialties in medicine [39]. In conditions of diagnostic uncertainty, physicians need to tolerate clinical ambiguity and evaluate risks and benefits of treatment in making decisions regarding information, communication, and escalation of care. The ability to tolerate uncertainty has been linked to the degree that physicians practice defensive medicine (to protect themselves from malpractice claims rather than to benefit the resident). In current theorizations of medical decision making, it is postulated that physicians make these decisions based on two distinct but related processes Type 1 (intuitive and affect-based) and Type 2 processes (deliberative and analytical) [17, 58]. This suggests that their affective experience (e.g. how they feel), beyond an evaluation of risks and benefits influences decision making [38, 56].

This influence of diagnostic uncertainty, perception of risk and practice of defensive medicine has not been studied in relation to nurses despite the significant role they play in RACF. Educational interventions in aged-care stewardship that address diagnostic uncertainty have targeted staff knowledge through dissemination of specific aged-care guidelines, passive and printed materials, active educational meetings/groups or audit and feedback for prescribers [21, 22, 46]. These interventions assume that inappropriate prescribing is driven by lack of knowledge in individuals. Increased knowledge and awareness of AMR has the potential to create dissonance between an individuals’ beliefs (based on that knowledge acquisition) and an individual’s behaviours. However, the lack of significant change in prescribing practices in RACF, despite development of educational interventions in the last decade, suggests that increasing knowledge *alone* is not enough. Therefore, the current review has two aims: firstly, to identify the attitudinal domains that have been measured in the AMS literature and comment on how these attitudes have been measured for all healthcare providers in RACF’s. Secondly, to consolidate our understanding of staff attitudes towards AMR and AMS in RACF. It is expected that findings will help in developing AMS interventions that target management of uncertainty and risk inherent in RACF settings.

## Method

### Search strategy and selection of studies

**Fig. 1:**
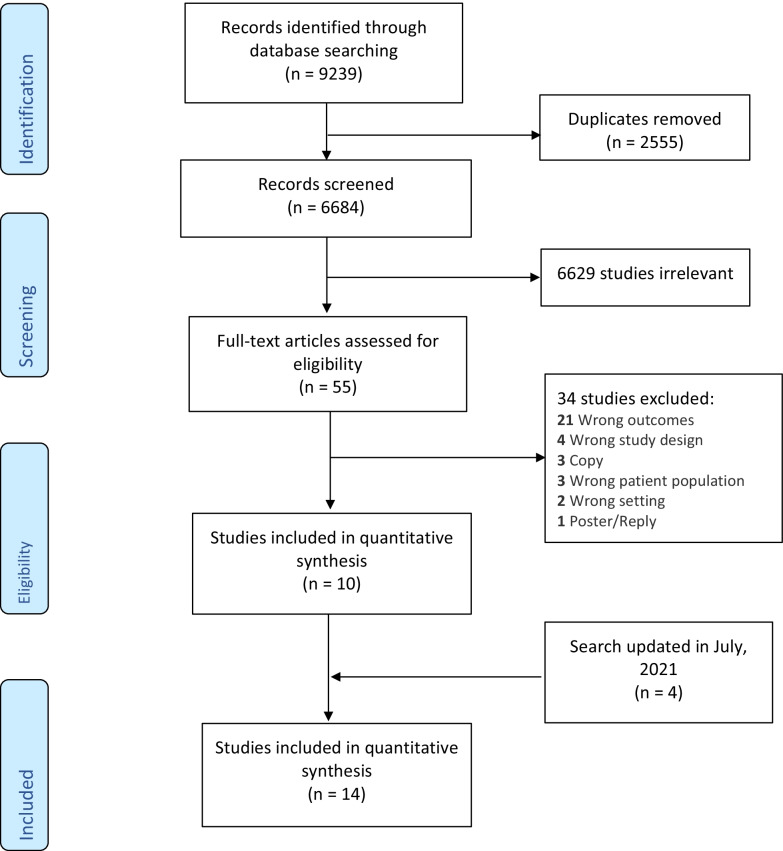
PRISMA flow chart

The review protocol was registered with PROSPERO: CRD42020184042. Following consultation with a librarian, a database search was conducted in August 2019, and updated in July 2021. Eight electronic databases—PsycINFO, PsycARTICLES, CINAHL Plus, MEDLINE, PubMed, Web of Science, Cochrane, and Scopus databases were searched for peer-reviewed empirical studies in English from the period of 1990-July 2021 (Additional file 1: Table S5). Types of documents included were research articles. Posters, replies to journal articles and unpublished thesis’ were excluded. Figure 1 shows the PRISMA flow chart of the number of articles identified across the databases. All searches were imported into Covidence, and duplicate articles were removed.

### Inclusion criteria

For the purposes of this review a broad definition of attitudes was used. Attitudes were defined as having three evaluative bases: cognitive, behavioural, and affective [65]. The cognitive components of attitudes consist of one’s thoughts or ideas, expressed as beliefs; behavioural components are expressed as observable behaviour or intention to act; and affective components consist of feelings or emotions that individuals experience regarding an attitude object [48]. For an article to be included the following criteria was to be met:

1. Participants had to be working in residential aged-care facilities (RACF’s) in some capacity (e.g. residential aged-care, long-term aged-care, veteran homes). Only settings where participants were responsible for the care of residents in a longer-term facility were included (acute or hospital settings were not included).
2. Studies must be conducted and written in English. Studies had to measure perspectives, beliefs or attitudes of participants.
3. Participants included doctors, nurses, nursing assistants, pharmacists and administrators, such as care managers.
4. The aged care nursing workforce is made up of various levels of nursing staff. The nurses can be broken up into three Levels. Level 1: Registered Nurses (at least a Bachelor’s degree) Level 2: License Practical Nurses (US) and Enrolled Nurse (Aus) (a TAFE style diploma) Level 3: Healthcare assistants, Assistants in Nursing and Personal Care Assistants (minimal training). In Australia and the US, the majority of the aged care workforce consists of level 3 nurses. In Australia, 66 percent of level 3 nurses hold a certificate III or higher in a relevant direct care field. In the US, less than half of the level 3 nurses have completed any formal education beyond high school [1, 2, 7, 8, 16, 49].
5. Studies needed to measure attitudes in relation to either prescribing antibiotics, resistance to antibiotics or antimicrobial stewardship.

Articles were initially screened by SS and DF based on the title and abstract, and any ineligible articles removed. At this level of screening, inclusion was liberal; articles were screened for studies based on criteria (a) and (b). Same authors then screened the full text of the articles for eligibility, with disagreements (n = 21) resolved by discussion where possible. Where conflict in ratings could not be resolved, the ratings of a third rater (FD) were used and final decisions were made through consensus.

### Data extraction

Following screening, data was extracted into an Excel (MS Office) sheet. Information extracted included study design, study setting, number of participants, mean age, gender, whether attitudes were measured explicitly or implicitly, other outcomes that were measured and the type of outcome that were measured (qualitative vs. quantitative). Quantitative data: The protocol for quantitative data extraction was finalized in consultation with FD and SS. Qualitative data: The protocol for qualitative data extraction was finalized in consultation with CD and SS, not included in the current article.

### Risk of bias assessment

Risk of bias assessments were undertaken using the National Institute of Health Tool for Before-After (pre-post) Studies with No Control Group and Observational Cohort and Cross-Sectional Studies scale [44, 57]. Studies were assigned a quality label by SS and DF depending on their rating across the quality criteria. The number of items scored as ‘high’ quality was divided by the total number of items rated (as not all items were relevant to all studies due to design). Studies were assigned a rating of ‘poor’ if less than 60% of the quality criteria were met; ‘fair’ if between 60 and 80%, and ‘good’ if above 80% of the criteria were met. Any conflicts were resolved through discussion.

### Underlying theory

Assessment of whether a theoretical framework was used in the study was completed by the author SS. Studies where no theory was discussed were assigned a “no” and studies where a theoretical framework had been considered for all constructs and explicitly discussed in the paper were assigned a “yes”. Studies where theory was used but only in relation to one or more constructs but not all constructs were assigned “somewhat”.

## Results

The search resulted in 9239 articles. Removing duplicates brought the total number of articles to 6684. After title and abstract screening, 49 articles were identified for full-text screening, 13 of which met the inclusion criteria for qualitative analysis and 10 met criteria for quantitative analysis. 4 additional articles met criteria when the search was updated in July 2021. This review focused on the quantative studies; qualitative studies will be analysed and reported separately elsewhere due to the heterogeneity of data across these studies. See Fig. 1 for the PRISMA flow chart.

### Study characteristics

Table 1 summarizes the characteristics of the included studies. Fourteen studies were included in the quantitative review. Studies came predominantly from the United States of America (n = 11), followed by Australia (n = 1), Belgium (n = 1) and France (n = 1). Five studies used an inter-professional sample of nurses and prescribers, six studies recruited nurses only, one study recruited medical coordinators (prescribers) only, one study recruited care managers and pharmacists and one study recruited social workers. The eligible studies included eleven cross-sectional designs and three pre-post educational interventions. Additionally, one study included semi-structured interviews, one used additional case-vignettes, one used discrete choice experiments with clinical scenarios and one included open-ended questions (qualitative data not included in current review).

**Table 1:** Study characteristics

Author (year)	Sample size	Location	Staff targeted	Study design	Additional components of study
Ahouah et al. [3]	109	France	Level 1 nursing staff	Cross sectional self-report survey	
Beeber et al. [9]	881	USA	Level 1 nursing staff	Cross sectional self-report survey	Discrete choice experiments with case vignette
Drekonja et al. [19]	534	USA	Level 1, 2 and 3 nursing staff and physicians	Cross sectional self-report survey	
Gahr et al. [25]	592	USA	Prescribers, nurse practitioners (NPs), DONs (Director of Nurses), and ICPs	Cross sectional self-report survey	
Hale et al. [27]	140	USA	Level 1 and 2 nursing staff	Pre-post self-report survey with an intervention	Educational intervention
Kidd et al. [32]	39	Belgium	Medical Coordinators (prescribers)	Cross-sectional self-report survey	
Jump et al. [28]	71	USA	Physicians, NPs and physician assistants	Pre-post self-report survey with an intervention	Educational intervention
Kistler et al. [35]	30	USA	Level 2 and 3 nursing staff and Prescribers	Cross-sectional self-report survey	Semi-structured interviews
Kistler et al. [33]	31	USA	Level 1, 2 and 3 nursing staff	Cross-sectional survey self-report	Case vignettes
Lacey [37]	34	USA	Social workers	Cross sectional self-report survey	
Pringle et al. [50]	24	Australia	Care managers and Pharmacists	Cross-sectional self-report survey	
Scales et al. [51]	31	USA	Level 1 and 2 nursing staff, DONs, infection control practitioner (ICP), and prescribers	Cross sectional self-report survey	Open ended questions
Wagner et al. (2019)	180	USA	DONs	Cross sectional self-report survey	
Wilson et al. [63]	103	USA	Level 1 and 2 nursing staff	Pre-post self-report survey with an intervention	Educational intervention

### Study quality and risk of bias

Study quality is included in Additional file 1: Table S1. The ratings of quality for studies were primarily ‘poor’ (twelve studies) or ‘fair’ (two studies). Several studies did not consider the impact of confounding variables or attempt to control for these variables in their analyses. Most studies did not demonstrate if exposure variables were clearly defined, valid and reliable across all study participants.

### Attitudinal domains

Ten domains of importance were identified across the studies. These domains broadly mapped into three categories of attitudes: attitudes related to stewardship, attitudes related to self and attitudes related to others (Table 2). There was significant variability in which domains were measured in each study and how these were measured across the studies. There is a lack of consensus on what is important in measuring the attitudes of RACF staff towards antibiotic prescribing and stewardship. Most studies described using existing literature to derive items, however it was unclear what theoretical models (if any) were used. Seven studies did not consider underlying theory, six studies considered underlying theory somewhat (e.g. reported deriving attitudinal domains from literature reviews) and only one study explicitly discussed underlying theory. Some studies indicated that the constructs were derived from elements of Theory of Planned Behaviour [51], Donabedian’s structure outcome (SPO) model and Contingency theory [30]. However, there was a missing theoretical framework to define *what* attitudinal constructs were of importance to stewardship and *how* these attitudes could be measured for most studies. As such, these constructs are ambiguous in capturing *distinct* attitudinal categories and findings need to be interpreted cautiously.

**Table 2:** Attitudinal domains

Stewardship			Self					System/other	
Antimicrobial prescribing	Guidelines	Educational interventions	Self-confidence regarding clinical assessment and prescribing	Awareness of AMR as a problem and Stewardship as a priority	Self-efficacy (perceived behavioural control)	Perception of role	Perception of risk (self and others)	Team culture and social norms	Patients and family member expectations

### Measurement of attitudinal domains

The measures for each construct and study are recognised in Additional file 1: Tables S2 and S3. The review identified four measures for guidelines and perception of role, three for educational interventions, five for awareness of AMR as a problem, and six measures for residents and family members’ expectations. The psychometric properties for these measures were not reported. Four measures were identified for quantifying attitudes towards antimicrobial prescribing and only Scales et al. [51] reported good internal consistency for the measure developed. Four measures were developed for self-confidence regarding clinical assessment and prescribing and only Drekonja et al. [19] reported good internal consistency for their measure. Three measures were developed to quantify self-efficacy, with Scales et al. [51] reporting good consistency for the same. Perception of risk was measured by six measures and only one study reported poor internal consistency for the measure used by Drekonja et al. [19] and derived by Trautner et al. [59]. Seven measures were identified to quantify team culture and Drekonja et al. [19] reported psychometric properties with acceptable internal consistency and good test–retest reliability and Wagner et al. (2019) reported at least acceptable consistency on all of the sub-scales.

Nine studies used attitude questionnaires alone, one study used clinical vignettes only, three studies used both and one study used retrospective data in combination with questionnaires. Where questionnaires were used, responses were rated on Likert scales and items were added to obtain scores for each scale. Gahr et al. [25] used only yes/no responses to all items measuring each of the constructs. Out of the forty-six measures across the ten domains, only three measured affective or emotional components of attitudes. Seven studies measured behaviour, while the majority of the studies measured cognitions or beliefs (Table 3).

**Table 3:** Attitudinal components

Domain	Components of attitudes		
	Emotions (affective response)	Beliefs (thoughts, beliefs)	Behaviours (tendency to act)
Antimicrobial prescribing		Kistler et al. [33] Hale et al. [27] Scales et al. [51]^a^	Lacey et al. [37]
Guidelines		Drekonja et al. [19] Kidd et al. [32] Gahr et al. [25]	Beeber et al. [9]
Educational Interventions		Kidd et al. [32] Gahr et al. [25] Hale et al. [27]	
Self-confidence regarding clinical assessment and prescribing	Hale et al. [27]	Wilson et al. [63] Jump et al. [28] Drekonja et al. [19]	
AMR as a problem/ AMS as a personal priority		Wilson et al., [63] Kidd et al. [32] Pringle et al. [50] Jump et al. [28] Hale et al. [27]	
Self-efficacy (perceived behavioural control)	Scales et al. [51] Hale et al. [27]	Wilson et al. [63] Jump et al. [28] Scales et al. [51]	
Perception of role		Pringle et al. [50] Kidd et al. [32] Wilson et al. [63] Ahouah et al. [3]	Pringle et al. [50]
Perception of risk (self and other)		Kistler et al. [33] Drekonja et al. [19] Hale et al. [27] Wilson et al., [63] Kistler et al. [35]	Beeber et al. [9] Drekonja et al. [19] Hale et al. [27] Kistler et al. [35] Kistler et al. [33]
Team culture/social norms	Drekonja et al. [19]	Wilson et al., [63] Scales et al. [51] Gahr et al. [25] Drekonja et al. [19] Wagner et al. (2019) Lacey [37] Jump et al. [28] Kidd et al. [32]	Drekonja et al. [19]
Perceptions of residents and family members expectations		Wilson et al. [63] Scales et al. [51] Gahr et al. [25] Hale et al. [27] Ahouah et al. [3]	Beeber et al. [9]

^a^Not all items available. From sample items available, it was concluded that only cognitions were measured

### Stewardship

#### Attitudes towards antimicrobial prescribing

This domain refers to studies that attempted to capture aged-care staff attitudes towards use of antibiotics; when antibiotics were not indicated; and the side effects of unnecessary antibiotic prescribing. As Table 1 shows the studies included in this review surveyed a broad range of the RACF staff. Scales et al. [51] reported that both, nurses and prescribers endorse beliefs in support of reducing antibiotic use in nursing homes, however, prescribers were significantly more supportive of reducing antibiotics (M = 6.1) compared with nurses (M = 5.7) on a 7-point attitude scale. Similarly, Lacey et al. [37] reported that lower percentages of medical directors (41%) encouraged use of antibiotics in end-stage dementia compared with directors of nursing (59%) and administrators (57%). Kistler et al. [34] indicate that the majority of nurses (71%) were aware of optimal use of antibiotics but despite this, 39% agreed that they expected an antibiotic if they were sick enough to see a doctor themselves. Additionally, Hale et al. [27] found that nurses’ reported antibiotics to be associated with perceptions of high quality care (M = 3.3, on a 5-point scale) and low likelihood of side-effects, such as rash (M = 2.8) and allergic reactions (M = 2.8). These findings indicate that aged-care staff are aware of when antibiotics are not indicated but endorse positive or favourable attitudes towards antimicrobial prescribing.

#### Attitudes towards guidelines

Findings from studies that measured attitudes towards guidelines suggest that RACF staff are aware of guidelines and endorse these as being important (15; [32, 25, 9]. However it is unclear in these reports how the participants feel about the application of these guidelines and how this influences implementation. For example, 3 out of 4 studies reported that majority of RACF staff expressed beliefs in favour of the use of AMS guidelines [19, 32, 25]. Yet Kidd et al. [32] also reported that only 34% of prescribers indicated that implementation of local guidelines would be useful for future stewardship projects (M = 2.9 on 5-point scale). Similarly, only 46% of these same participants felt complimentary investigations guidelines would be useful in future (M = 2.9). Similarly, Drekonja et al. [19] reported a discrepancy between staff agreement with statements about the importance of AMS guidelines (M = 4.2, 5-point scale) and the AMS-related behaviour scores (M = 3.4) reported in corresponding vignettes. Beeber et al. [9] attempted to measure nurses’ behaviour (the likelihood of calling the prescriber) based on symptoms for UTIs through use of discrete choice experiments in clinical vignettes. Symptoms such as painful urination (OR = 4.85, CI 4.16–5.65) and high temperature (OR = 3.80, CI − 3.28 to 4.42) had the highest importance scores for calling prescribers which were concordant with ‘evidence-based practice’ described in the study.

#### Attitudes towards educational interventions

Stewardship intervention in RACF have a strong educational focus on staff [64]. Yet only three studies in this review measured the attitudes of RACF staff towards these interventions [32, 25, 27]. Kidd et al. [32] found that there was stronger support for integrating teaching about antimicrobial use during medical training (90%) and for antimicrobial stewardship training of medical coordinators (79%). There was less support for basic training for nurses (56%) and online continuous education (41%) as useful interventions. This was in contrast with previous findings by Gahr et al. [25]that indicated that 62% physicians, 72% nurse practitioners and 73% of other health staff agreed that there was a need for education of nurses. Hale et al. [27] reported that while the majority of nurses felt positively towards the relevance and likelihood of application of the AMS learning modules used in the study, approximately 5–9% endorsed beliefs that they were unlikely to apply the learning modules in their daily practice. These findings suggest that even though giving a greater role to non-prescribers in AMS in RACF is seen as providing great benefits, prescribers are still seen as being the primary focus for antimicrobial stewardship.

#### Self-confidence regarding clinical assessment and prescribing

Four studies measured the confidence of RACF staff regarding their knowledge about clinical assessment and appropriate prescribing (Jump et al. 2015; [19, 63, 27]. All three studies found that staff reported relatively high levels of confidence, however it was unclear if educational interventions improved confidence. Wilson et al. [63] focused on staffs’ beliefs about their ability to differentiate between the symptoms of a urinary tract infections (UTI) and asymptomatic bacteria ASB) and reported a significant improvement in respondents’ confidence after an educational intervention (pre-intervention M = 3.9; post-intervention M = 4.2, *p* < 0.05). In comparison, Jump et al. [28] focused on whether the staff was able to differentiate between the causes of an infection (i.e. whether staff were able to determine if pneumonia was caused by bacteria or virus). Similar to Wilson et al. [63] staff reported high confidence ratings (> 70%) on all items except being able to determine if pneumonia is caused by bacteria or a virus [28]. Notably the studies by Jump et al. [28] and Hale et al. [27] found no significant differences in the confidence of their participants in recognising symptoms of a UTI or acute respiratory tract infection (ARTI) after an educational intervention. In contrast to previous studies, Drekonja et al. [19] measured and reported that most prescribers felt positively (M = 4.2) regarding knowing when to order a urine culture, how to manage bacteriuria in a patient and being able to apply guidelines to patients.

#### AMR as a problem and stewardship as an individual priority

Six studies measured participants’ awareness of AMR; and/or consideration of stewardship as a personal priority [63, 32, 50, 25, 28, 27]. All six studies found that RACF staff (nurses, prescribers) and associated healthcare staff (pharmacists and care managers) were aware of AMR. Although 87% of RACF staff are aware of multi-drug resistance in their practice, 52% considered infection prevention to be more important than antimicrobial stewardship [28]. Further, there were no significant changes in the belief regarding infection control being more important after an educational intervention (pre-test M = 3.6, post-test M = 3.8; *p* > 0.05) [63].

#### Self-efficacy/perceived behavioural control

This domain refers to studies that attempted to capture staff’s self-efficacy or perceived behavioural control in driving antimicrobial stewardship in RACF [63, 51, 28, 27]. All studies found that aged-care staff endorsed beliefs reflecting high self-efficacy. Hale et al. [27] found nurses reported high confidence in their ability to contact prescribers to discuss infection symptoms (M = 4.5, 5-point scale) and explain to resident/family why antibiotics are not necessary (M = 4.2) and these ratings did not change after the educational intervention. Similarly, Wilson et al. [63] also found that nurses reported high levels of self-efficacy (M > 4.0 on all items) and that there was a significant increase in nurses’ ability to tell whether changes (to a patient’s clinical status) were due to an infection after the educational intervention (pre-test M = 4.0; post-test M = 4.5; *p* < 0.05). Only Scales et al. [51] compared self-efficacy ratings across professional groups by using sub-scales for change commitment, efficacy and readiness for change sub-scales (see Additional file 1: Table S2). They found that nurses reported significantly higher change commitment (M = 4.1 nurses; M = 3.9 prescribers, *p* < 0.05) and change-efficacy ratings (M = 4.0 nurses; M = 3.9 prescribers, *p* < 0.05) compared with prescribers. Nurses perceived their own group readiness for change to be similar to prescribers’ (M = 3.6 for nurses and M = 3.8 for prescribers), but medical provider’s ratings of group readiness for change in nurses was lower (M = 2.9 for nurses versus M = 3.8 for prescribers).

#### Perception of role

Findings from studies suggest agreement among nurses’ regarding their role in stewardship. 85.3% of nurses perceive themselves as being sources of information for patients regarding antibiotics [15], and agreed (5-point scales) that their assessment of the patient (M = 3.8), communication with providers (prescribers) (M = 4.1), knowledge of a patients’ baseline (M = 3.8), clinical assessment of a patient and communication with patient and family members (M = 3.8) influenced whether the patient received antibiotics [63]. However, it is unclear how other RACF staff (e.g. prescribers, nursing assistants) perceive their roles in AMS. Findings from Kidd et al. [32] indicate that general practitioners (GPs) perceive other aged-care staff, such as nurses (M = 3.5), medical coordinators (M = 3.1), and hospital specialists (ID or AMS team) (M = 3.1) to have a larger role in stewardship compared with themselves (M = 2.9)”.

#### Perception of risk

This domain was defined in two ways: risk to self (e.g. risk of the participant getting worse when sick themselves or professional risk e.g. de-registration, litigation); and risk to others (e.g. residents suffering serious side effects or death due to stewardship strategies such as guideline-adherent prescribing). Only one study measured perceptions of risk to self and reported that 6% of nurses agreed with the statement that they should take antibiotics to prevent serious illness [33]. No studies assessed the implications of professional risk for not prescribing antibiotics and a resident’s illness becoming worse or resulting in death.

Six studies measured perceptions of risk to others. Four of these studies used a sample of nurses and produced mixed findings. Wilson et al. [63] used a single item to measure nurses agreement (M = 4.1) with the belief that it was reasonable to monitor a resident in conditions of uncertainty over providing antibiotics for a period of 1–2 days. Kistler et al. [33] used three clinical vignettes to measure nurses agreement with the prescribing of antibiotics for a wound (3%), viral upper respiratory tract infection (URTI) (13%) and asymptomatic bacteriuria (ASB) (23%). Findings from these studies suggest that the majority of nurses agreed that it was appropriate to wait in cases of uncertainty and that antibiotics were not indicated for all three vignettes. Similarly, Hale et al. [27] also used vignette style questions to measure nurses risk perceptions and found that the majority of nurses incorrectly perceived that signs (e.g. bacteria in urine) ‘sometimes’ indicated the need for antibiotics. The study concluded that an educational intervention was useful in reducing these scores in specific areas, such as foul-smelling urine (pre-test M = 2.7; post-test M = 2.1; *p* < 0.05). Similar to these findings, Beeber et al. [9] also found that criteria, such as history of UTIs and urinalysis that was not indicated to be ‘evidence-based’ contributed to decisions regarding calling prescribers for a suspected UTI.

Two of the studies compared an interprofessional sample of RACF staff. Kistler et al. [35] found differences in risk perceptions between prescribers and nurses. The study reported although all prescribers had prescribed an antibiotic for the resident, only 40% felt it was “not at all likely” that the resident would have gotten better without the medication compared with 78% of nurses. These findings suggest that in general, prescribers are more likely to accept the risk of overprescribing antibiotics compared to nurses who tend to support conservative prescribing practices in aged-care. Drekonja et al. [19] did not provide participant ratings for individual items or comparison for risk perceptions across professional groups, but reported lower risk perception (M = 3.8) and behaviour scores (M = 3.4) compared to guideline acceptance (M = 4.2) and self-efficacy scores (M = 4.2) for prescribers. Further, they also found a significant correlation between risk perceptions and prescribing behaviour (*P* = 0.04 and *P* = 0.02). These findings further support the discrepancy between self-reported beliefs and behaviours regarding stewardship.

#### Social norms and team culture

Eight studies examined social norms and team culture, with significant variability in the measures used. Several studies measured the perception of influence of different professional groups in prescribing and findings suggest that there are inter-professional tensions that warrant further investigation. Although nurses are perceived to be the drivers of resident care by prescribers [32], social workers reported medical directors (45%), directors of nursing (25%) and administrators (10%) rather than nurses to be perceived as the most influential in driving resident care [37]. Furthermore, several studies that examined RACF staff perceptions of inter-professional relationships had mixed findings. Gahr et al. [25] found that although only 9.2% of physicians reported communication between nurses and physicians to be a problem, 56% physicians and 50% of nurse practitioners agreed that perceived pressure from nurses to order urine cultures contributed to antibiotic overprescribing. Similarly, Wagner et al. (2019) found that the majority of staff reported good interprofessional relationships in RACF’s, 33.1% of nurses did not perceive physicians to be committed to antibiotic stewardship and 28.3% of physicians were not perceived to have a good relationship with licensed nurses and 10.2% of nursing directors agreed that nursing staff ‘get no respect from physicians’. Similar findings were reported by Scales et al. [51] regarding prescribers perceptions of nursing staff’s readiness for change (M = 2.9) to be lower than their own (M = 3.8).

Drekonja et al. [19] used an established measure—the Safety Attitudes Questionnaire (SAQ) Short Form [53] to deduce a ‘teamwork’ and ‘safety climate’ (defined as healthcare staff’s attitudes towards patient safety) score. The study reported significant differences in both, safety climate scores and teamwork scores across professional groups (calculated using the formula: (mean scores of items belonging to the scale − 1) × 25). Clinical Nurses Assistants (CNA’s) reported highest safety scores (M = 77.5, 100-point scale) followed by staff providers (experienced prescribers) (M = 71.2), nurses (M = 69.1), and then resident prescribers (M = 64.7). Similarly, CNA’s had highest teamwork scores (M = 76.3), followed by resident prescribers (M = 71.2), nurses (M = 70.7) and resident prescribers (M = 65.1). Further, teamwork climate was also found to be significantly correlated with social norms (*P* = 0.04).

#### Family members and resident expectations

RACF staff perceptions of residents and family members’ expectations has been explored in several studies using an inter-professional sample (prescribers, nurses, administrators and social workers). Beeber et al. [9] reported that the majority of nurses agree that antimicrobial requests from residents and their family members should not initiate calls to prescribers for antibiotics. However, despite these beliefs most studies concluded that RACF staff perceive that residents and their families: (1) expect antibiotics when there is suspicion of an infection; and, (2) believe that antibiotics are associated with high quality care and that no changes in these perceptions were found after educational stewardship interventions [63, 51, 27, 3, 37]. Additionally, a study that asked respondents to identify the staff in facilities that have the most influence regarding medical interventions for residents, 20% responded “other”, with many specifying family members as most influential under this category [37].

Two studies compared nurses and prescribers’ perceptions and both studies found that significantly more nurses than prescribers endorsed the belief that antibiotics are expected and that family members influence prescribing decisions [51, 25]. Perhaps reflecting the effect of being involved in the day-to-day care of residents, Scales et al. [51] reported that while both nurses (M = 3.4) and prescribers (M = 3.1; *p* < 0.05) endorsed the belief that family members and residents’ had a preference for antibiotics. Nurses’ rated the influence of family members (M = 2.8) and residents’ on prescribing significantly higher than prescribers (M = 2.4; *p* < 0.05).

## Discussion

Despite the significant push towards enhancing AMS in RACF over the last decade, this review identified significant gaps in our understanding of healthcare staff attitudes towards stewardship (Additional file 1: Table S4). Most importantly, the attitudinal domains examined were quite diverse and there is a lack of theory to drive clarification of the constructs and their measurement. Secondly, important domains, such as staff perceptions of professional risk for reducing antibiotics have not been assessed in any of the studies. A number of studies identified inter-professional tensions in how nurses and other staff perceive nurses role in influencing antibiotic overprescribing [63, 51, 27, 32, 3, 37]. There were conflicting findings regarding the efficacy of educational interventions in shifting beliefs, self-confidence, and awareness of AMR [63, 32, 25, 28, 27] despite the continued emphasis of educational interventions to address stewardship in RACF.

There is not always a great deal of clarity about what the specific attitude domain is for some measures is (e.g. attitudes are sometimes assumed based on individuals confirming that they would engage in particular behaviours, inferring, for example, participant attitudes towards prescribing are based on responses such as “I would call the doctor….”). There is confusion between the measurement of knowledge and attitudes due to these being poorly defined within the stewardship literature. Our results are consistent with the findings of Beeber et al. [9] that the vast majority of AMS interventions try to fill knowledge gaps rather than understanding how healthcare staff feel about stewardship. The development of multiple new measures in studies with the provision of minimal psychometric data made it difficult to make conclusive statements about the quality of data that has been measured (e.g. only 13% of measures reported psychometric properties). There was an emphasis on measurement of beliefs; and some studies collapsed the measures of behaviour when creating study variables. Moreover, the majority of studies did not assess staffs’ affective experience across the constructs identified to be of importance to AMS. Most studies reported that staff positively endorse guideline-adherent beliefs regarding antimicrobial prescribing. However, there is a discrepancy between their acceptance of such guidelines and behaviours that contribute to overprescribing in conditions of uncertainty (e.g. identifying the cause of an infection) [63]. Previous findings in adjacent literature highlight that healthcare staff often feel anxiety related to diagnostic uncertainty [39]. Therefore, it is likely that healthcare staff’s attitudes towards stewardship are also influenced by their affective, evaluative and behavioural processes in a reciprocal fashion [4]. These findings point to the importance of measuring staff’s affective experience in addition to their cognitions and behaviours.

Despite the limitations of the attitudinal constructs identified above, there were a number of important findings from the current review. Key among these is that factors related to the healthcare staff’s environment (e.g. family members’ expectations and perception of role) contribute towards staff attitudes towards stewardship. The tensions between the individual’s perception of their role in stewardship and how their role might be perceived by others (e.g. family members, other aged-care staff) in the system seems to play a significant role in influencing behaviour. In general, most staff have positive attitudes towards and believe they have a role in reducing antibiotic use, but nurses were perceived to be less willing to reduce antibiotic use compared to physicians (50, 51, 25). Despite the significant role that nurses play in aged-care, residents do not perceive nurses to be the source of information for antibiotics [3] and other healthcare staff (such as social workers) perceived prescribers to be the most influential in driving decisions about resident care in RACF [37]. Although research is limited, most nurses (76%) were positively disposed to educational interventions to address AMR, but far fewer (< 53%) other health care staff believed such training was needed for nurses [25]. Given the high levels of resident contact and the key role they play in assessment and communication of resident health status, it is somewhat surprising that such discrepancies in attitudes are present.

A number of studies also identified differences between nurses’ and prescribers perceptions of pressure for prescribing antibiotics. Nurses’ ratings of the degree of influence that the resident and family members’ exert on prescribing was significantly higher compared to the influence that prescribers said they felt from residents’ and their family members [51] and nurses also perceived antibiotic prescribing to positive influence perceptions of high-quality care [27]. Prescribers also reported experiencing pressure from nurses [25]. Given that nurses are frequently the primary contact for residents (and families) it is possible that nurses inadvertently pass on the pressure they feel from residents and family members to prescribers. This requires further exploration, particularly for Level 3 nurses that are closest to interacting with residents and their family members on a daily basis.

Another key finding is that individual factors such as risk perceptions have been identified to be a significant barrier to reducing overprescribing [5]. It is well-established that prescribing antibiotics over the phone in aged-care settings remains a common practice, even under conditions of uncertainty about resident symptoms and diagnosis [9]. The scholarly literature on medical decision-making identifies anxiety regarding risk of malpractice in influencing decisions [11]. In the aged-care settings, pressure from family and staff members have been noted as additional non-clinical risk factors in making clinical decisions. However, despite the potential for these factors to contribute to over-prescribing, none of the studies have assessed staff attitudes towards risk to self (e.g. litigation, de-registration). Some studies have measured the perceived risk to residents (e.g. worsening symptoms, death) and found significant differences in risk perception between non-prescribers and prescribers. Risk perceptions have also found to be correlated with teamwork, social norms and safety climate [19, 35]. Given the potential impact of perception of risk on medical decision-making, this warrants further attention in RACF settings.

Finally, our findings suggest that factors other than a theoretical knowledge of prescribing might play a crucial role in contributing towards overprescribing. Educational interventions appear to be useful in addressing specific knowledge gaps such as differentiating and interpreting different signs and symptoms [63], but do little to increase staff’s confidence in navigating psychosocial barriers identified above [27].

The current review has several limitations. There is large variability in practices in aged-cares, how stewardship interventions are designed and measured as well as how overprescribing is measured. Meta-analysis was not conducted due to relatively small number of studies identified and the heterogeneity of study designs. Most studies presented in the current review were cross-sectional or pre-post surveys in design, limiting the ability to make any causal statements regarding the role of attitudes on stewardship activities. In addition, the studies did not measure changes in attitudes over a longer period of time. Future research should consider clearly defining each attitudinal domain, with particular attention to attitudes towards risk perception, self-efficacy, and perception of role and family members’ expectations. Systematic reviews of stewardship in aged-cares have conceptualized overprescribing as a result of both, lack of knowledge and problem awareness in healthcare staff, especially nurses. However, the findings from the current review suggest that cognitive awareness of the problem of antimicrobial resistance and guideline-adherent prescribing *alone* is insufficient. Consideration of the influence of the affective experiences of staff in their perception of risk, perception of role and self-efficacy; team culture and others’ expectations in escalating resident care is needed. This can be done through the use of a combination of case-vignette style questionnaires and explicit items asking staff regarding their cognitive process (e.g. worries regarding de-registration or litigation).

## Conclusion

Stewardship in RACF have been driven with a focus towards educational interventions to address the assumption that overprescribing occurs as a result of knowledge gaps [9]. The primary focus for these interventions have been prescribers, with a more emerging focus on the nurses role in stewardship [14, 26]. This review highlights that there is a lack of theory guiding the measurement of healthcare staff’s attitudes regarding stewardship. There is little consensus regarding which attitudinal domains are of importance to stewardship and how these can be measured. Particularly little attention has been given to the conceptualization of attitudes and the psychometric properties of the measurements used. The overemphasis on measuring attitudes through the self-report of beliefs limits our understanding of how healthcare staff feel about stewardship strategies and management of non-clinical risk factors (e.g. risk, perceptions of role and expectations of family members) and how this effects their behaviours and in turn plays a role in antibiotic overprescribing. Further research is needed to address these gaps and deepen our understanding of the discrepancy between staff-reported beliefs regarding AMS and implementation of behaviours that support AMS in RACF.

## Supplementary Information

**Additional file 1.** Supplementary Tables.

## Data Availability

All data generated or analysed during this study are included in this published article [and its Additional file 1].
