# Supplementary material for: How do aged-care staff feel about antimicrobial stewardship? A systematic review of staff attitudes in long-term residential aged-care
Source: Antimicrob Resist Infect Control. 2022 Jun 28;11:92. doi: 10.1186/s13756-022-01128-5 (PMC9238058; doi:10.1186/s13756-022-01128-5)
Supplement: Supplementary file 1 — Additional file 1. Supplementary Tables. [file 13756_2022_1128_MOESM1_ESM.docx]

**Supplementary Tables**

Supplementary Table 1: Study Quality

| **Author (year)** | Wilson et al., (2017) | Kistler et al. (2013) | Kid et al. (2016) | Pringle et al. (2016) | Kistler et al. (2017) | Scales et al. (2017) | Gahr et al. (2007) | Jump et al. (2015) | Hale et al. (2017) | Drekonja et al. (2019) | Beeber et al. (2021) | Ahouah et al. (2019) | Lacey (2005) | Wagner et al. (2019) |
| --- | --- | --- | --- | --- | --- | --- | --- | --- | --- | --- | --- | --- | --- | --- |
| **Study quality** | Fair | Poor | Poor | Poor | Fair | Poor | Poor | Poor | Poor | Fair | Poor | Poor | Poor | Poor |

Supplementary Table 2: Measures

|  | **Construct** | **Items** | **Theory** | **Source of measure** | **Response format** | **Psychometric data (reliability, validity)** |
| --- | --- | --- | --- | --- | --- | --- |
| Ahouah et al. (2019) | Perception of Role | 1 | No | New | 5-point Likert scale with response options ranged from 1 (strongly disagree) to 5 (strongly agree) | Not reported. |
|  | Residents and family members expectations | 10 | No | New | 5-point Likert scale with response options ranged from 1 (strongly disagree) to 5 (strongly agree) | Not reported. |
| Beeber et al. (2021) | Residents and family members expectations | 2 | Somewhat – literature review, list of evidence based and non-evidence-based criteria used in decision making for UTIs (Kistler et al., 2020). | New | 4 response options: “I would not call the doctor at this time,” “I would call the doctor about a suspected UTI,” “I would call the doctor about something other than a suspected UTI,” and “I would call the doctor about multiple suspected infections, including UTIs. | Not reported. Pilot testing done with nurses online. |
|  | Perception of Risk (Self & Other) | 5 | Somewhat – literature review, list of evidence based and non-evidence based criteria used in decision making | New | 4 response options: “I would not call the doctor at this time,” “I would call the doctor about a suspected UTI,” “I would call the doctor about something other than a suspected UTI,” and “I would call the doctor about multiple | Not reported. Pilot testing done with nurses online. |
|  |  |  | for UTIs (Kistler et al., 2020) |  | suspected infections, including UTIs. |  |
|  | Guidelines | 24 | Somewhat – literature review, list of evidence based and non-evidence based criteria used in decision making for UTIs (Kistler et al., 2020) | New | 4 response options: “I would not call the doctor at this time,” “I would call the doctor about a suspected UTI,” “I would call the doctor about something other than a suspected UTI,” and “I would call the doctor about multiple suspected infections, including UTIs. | Not reported. Pilot testing done with nurses online. |
| Drekonja et al. (2019) | Guidelines | 1 | Somewhat – literature review using similar surveys in other clinical areas (e.g., management of hypertension). | Existing – modified (Trautner et al., 2014). | 6-point scale. Response options ranged from 1 (strongly disagree) to 5 (strongly agree) and (0) Don’t know. | Not reported |
|  | Self-Confidence regarding Clinical Assessment & Prescribing | 3 | Somewhat – literature review, unclear which specific psychological theories were  used to design the specific items used. | Existing - (Trautner et al., 2014). | 6-point scale. Response options ranged from 1 (strongly disagree) to 5 (strongly agree) and (0) Don’t know. | The Cronbach alpha for this scale was 0.82. |
|  | Perception of Risk (Self & Other) | 9 | Somewhat – literature review, unclear which specific psychological theories were  used to design the specific items used. | Existing - (Trautner et al., 2014). | 6-point scale. Response options ranged from 1 (strongly disagree) to 5 (strongly agree) and (0) Don’t know. | The Cronbach alpha for ‘risk perceptions’ was 0.53 and ‘behaviour’ was 0.56. |
|  | Team culture | 2 subscales (teamwork climate, safety climate), 13 items in total. 3 items in the ‘social norms scale’ | Yes – literature analysis and use of 2 conceptual models Vincent's framework for analysing risk and safety and Donabedian's conceptual model for assessing quality (Sexton et al., 2006). | Existing – modified. The Safety Action Questionnaire (short form) is used to measure organisational teamwork climate and safety culture (Sexton et al., 2006). 3 other items were added to measure social norms. | 6-point scale. Response options ranged from 1 (strongly disagree) to 5 (strongly agree) and (0) Don’t know. | The SAQ has been reported to have satisfactory fit (χ2 (784) = 10,311.27, p < .0001; CFI = .90, and strong reliability (ρ value =.90) (Sexton et al., 2006).  The Cronbach alpha for social norms was 0.63 with 4 items. |
| Gahr et al. (2007) | Guidelines | 1 | No | New | Yes/No response | Not reported |
|  | Educational interventions | 2 | No | New | Yes/No response | Not reported |
|  | Residents and family members expectations | 1 | No | New | Yes/No response | Not reported |
|  | Team culture | 2 | No | New | Yes/No response | Not reported |
| Hale et al. (2017) | AMR/AMS as a problem | 1 | No | Existing – Taken from the Minnesota Department of Health’s Antibiotics Stewardship Program Toolkit for Long-Term Care Facilities ("Minnesota Antimicrobial Stewardship Program Toolkit for Long-term Care Facilities - Minnesota Dept. of Health", Updated 2021) | 5-point Likert scale using (5) always to (1) never. | Not reported. The survey was evaluated for face validity using nursing and pharmacy students. |
|  | Residents and family  members expectations | 3 | No | Existing – Taken from the Minnesota Department of  Health’s Antibiotics Stewardship Program Toolkit for Long-Term Care Facilities (2021). | 5-point Likert scale using (5) always to (1) never. | Not reported. The survey was evaluated for face  validity nursing and pharmacy students. |
|  | Educational interventions | 3 | No | New | 4-point scale using (4) Very high quality to (1) Very low quality | Not reported. The survey was evaluated for face  validity nursing and pharmacy students. |
|  | Perception of Risk (Self & Other) | 22 | No | Existing – Taken from the Minnesota Department of Health’s Antibiotics Stewardship Program Toolkit for Long-Term Care Facilities (2021) | 5-point Likert scale using (5) always, (4) often, (3) sometimes, (2)  rarely, (1) never. | Not reported. The survey was evaluated for face validity using nursing and pharmacy students. |
|  | Self-Confidence regarding Clinical Assessment & Prescribing | 2 | No | New | 5-point Likert scale from (1) strongly  Disagree to (5) Strongly agree. | Not reported. The survey was evaluated for face validity using nursing and pharmacy students. |
|  | Self-efficacy (Perceived behavioural Control) | 3 | No | New | 5-point Likert scale from (1) strongly  Disagree to (5) Strongly agree. | Not reported. The survey was evaluated for face validity using nursing and pharmacy students. |
|  | Antimicrobial prescribing | 7 | No | Existing – Taken from the Minnesota Department of Health’s Antibiotics Stewardship Program Toolkit for Long-Term Care Facilities (2021) | 5-point Likert scale using (5) always to (1) never. | Not reported. The survey was evaluated for face validity using nursing and pharmacy students. |
| Jump et al. (2015) | AMR as a problem | 4 | No | New | Questions used a slider bar with a range of 1 to 100 to assess agreement with statements. | Not reported |
| Jump et al. (2015) | Self-efficacy (perceived behavioural control) | 1 | No | New | Questions used a slider bar with a range of 1 to 100 to assess agreement with statements | Not reported |
|  | Team culture | 1 | No | New | Questions used a slider bar with a range of 1 to 100 to assess agreement with statements | Not reported |
|  | Self-Confidence regarding Clinical Assessment & Prescribing | 5 | No | New | Questions used a slider bar with a range of 1 to 100 to assess agreement with statements | Not reported |
| Kidd et al. (2016) | Guidelines | 3 | Somewhat - literature analysis. | New | 5-point Likert scale from 0 (I do not agree at all) to 5 (I totally agree) | Not reported, pilot testing with infection  prevention specialists |
|  | Educational intervention | 4 | Somewhat - literature analysis. | New | 5-point Likert scale from 0 (I do not agree at all) to 5 (I totally agree) | Not reported, pilot tested with infection prevention specialists. |
|  | AMR/AMS as a problem | 6 | Somewhat - literature analysis. | New | 5-point Likert scale from 0 (I do not agree at all) to 5 (I totally agree) | Not reported, pilot testing with infection prevention specialists. |
|  | Perception of Role | 1 | Somewhat- literature analysis. | New | 5-point Likert scale from 0 (I do not agree at all) to 5 (I totally agree) | Not reported, pilot testing with infection prevention specialists. |
|  | Team culture | 3 | Somewhat – literature analysis. | New | 5-point Likert scale from 0 (I do not agree at all) to 5 (I totally agree) | Not reported, pilot testing with infection  prevention specialists. |
| Kistler et al. (2013) | Perception of Risk (Self & Other) | 2 | No | New | Staff ratings of severity of sickness and likelihood of recovery without antibiotics were measured using the following scale: (1) Not at all, (2) A little, (3) Moderately, (4) A great deal (5) Don’t Know. | Not reported |
| Kistler et al. (2017) | Perception of Risk (Self & Other) | 4 | Somewhat – literature analysis. | Existing – previously used (Vanden et al., 2003)  Newly developed clinical vignettes | 5-point Likert scale (5) strongly disagree to, (1) strongly agree.  Clinical vignettes were rated as yes/no responses. | Not reported. |
|  | Antimicrobial prescribing | 3 | Somewhat – literature analysis. | Existing – previously used (Vanden et al., 2003). Vignettes created for current study. | 5-point Likert scale (5) strongly disagree to, (1) strongly agree. | Not reported. |
| Lacey (2005) | Team culture | 3 | No | New | Scale with 4 descriptive options:  Don’t know; Takes no position; Encourages antibiotics (in the absence of advance directives and family is uncertain; Discourages the use of antibiotics except for comfort care. | Not reported. |
|  | Antimicrobial prescribing | 1 | No | New | Scale with 4 descriptive options:  Don’t know; Takes no position; Encourages antibiotics (in the absence of advance directives and family is uncertain; Discourages the use of antibiotics except for comfort care. | Not reported. |
| Pringle et al., (2016) | AMR/AMS as a problem | 3 | No | New | Yes/No question.  5-point Likert scale (5) strongly disagree to, (1) strongly agree. | Not reported. Pilot tested with 1 pharmacist and care manager. |
|  | Perception of Role | 2 | No | New | Yes/No question.  5-point Likert scale (5) strongly disagree to, (1) strongly agree. | Not reported. Pilot tested with 1 pharmacist and care manager. |
| Scales et al. (2017) | Team culture | 2 | Somewhat – literature analysis. | New | 5-point Likert scale. Response options ranged from 1 (strongly disagree) to 5 (strongly agree). | Not reported |
|  | Residents and family members expectations | 4 | Somewhat – literature review. | New | 4-point scale (1–4: never, sometimes, usually, always) | Not reported |
|  | Antimicrobial prescribing | 5 | Yes – informed by the “direct measure of attitude” from the | New | 7-point scale from (1) strongly disagree to (7) strongly agree. | The internal consistency (Cronbach alpha) for the items was 0.87 for medical |
|  |  |  | theory of planned behaviour items. |  |  | providers and 0.90 for nurses. |
|  | Self-efficacy (perceived behavioural control) | 12 | Yes – Weiner’s theory of organisational readiness for change (Shea et al., 2014). | Existing measure - Organizational Readiness for Implementing Change instrument (Shea et al., 2014). | 5-point Likert scale. Response options ranged from 1 (strongly disagree) to 5 (strongly agree). | Internal consistency reported to be high (Cronbach alpha ranged from 0.87 to 0.93) for each of the 2 subscales. |
| Wagner et al. (2019) | Team culture | 4 sub-scales, 11 items. | Yes - Donabedian’s structure/process/outcome (SPO) model and Contingency theory (Katz et al., 2009) | Existing – adapted from the survey of NH physicians, the NH Medical Staff Organization and Culture (NHMSO) (Katz et al., 2009) | 2 sub-scales used a 5-point Likert scale (1) Strongly disagree to (5) Strongly agree.  5-point Likert scale using (1) Not involved to Very involved. | The Cronbach alpha for the different sub-scales ranged from 0.61-0.81. |
| Wilson et al., (2017) | Self-Confidence regarding Clinical Assessment & Prescribing | 6 | No | New | 5-point Likert scale (5) strongly disagree to, (1) strongly agree. | Not reported |
|  | AMR /AMS as a problem | 4 | No | Existing (Jump et al. (2015)– modified | 5-point Likert scale (5) strongly disagree to, (1) strongly agree. | Not reported |
|  | Self-efficacy | 5 | No | Existing - Jump et al. (2015) | 5-point Likert scale (5) strongly disagree to, (1) strongly agree. | Not reported |
|  | Perception of Role | 4 | No | New | 5-point Likert scale (5) strongly disagree to, (1) strongly agree. | Not reported |
|  | Residents and family members expectations | 1 | No | New | 5-point Likert scale (5) strongly disagree to, (1) strongly agree. | Not reported |
|  | Team culture | 4 | No | Existing - Jump et al. (2015) | 5-point Likert scale (5) strongly disagree to, (1) strongly agree. | Not reported |
|  | Perception of Risk (Self & Other) | 1 | No | New | 5-point Likert scale (5) strongly disagree to, (1) strongly agree. | Not reported |

Supplementary Table 3: Synthesis of Psychometrics Used for Attitudinal Domains

| **Attitudinal Domain** | **Number of measures** | **Psychometrics reported** |
| --- | --- | --- |
| Antimicrobial prescribing | 4 | 1 |
| Guidelines | 4 | 0 |
| Educational interventions | 3 | 0 |
| Self-Confidence regarding Clinical Assessment & Prescribing | 4 | 1 |
| Awareness of AMR as a problem and Stewardship as a priority | 5 | 0 |
| Self-efficacy (perceived behavioural control) | 4 | 1 |
| Perception of Role | 4 | 0 |
| Perception of Risk (Self & Others) | 6 | 1 |
| Team | 7 | 2 |
| Family expectations | 6 | 0 |

Supplementary Table 4: Key Findings for Stakeholders in RACF

| **Domain** | **Nurses** | **Prescribers** | **Other Staff** | **Conclusions and**  **Further Recommendations** |
| --- | --- | --- | --- | --- |
| Attitudes towards antimicrobial prescribing | Less supportive of reducing antibiotic prescribing compared to prescribers (Scales et al., 2015).  Use of antibiotics associated with perceptions of high-quality care, lower rates of influenza and lower rates of side-effects (Hale et al., 2017) | More supportive of reducing antibiotic prescribing compared to nurses (Scales et al., 2015). |  | These findings suggest that exploring why nurses feel positively towards antibiotics and how nurses feel towards *reducing* prescribing is important for future research. |
| Attitudes towards Guidelines | Discrepancies between acknowledgement of guidelines being helpful and implementation of guideline-adherent behaviours (Beeber et al., 2021). | Guidelines perceived to be helpful but future implementation only supported by 34-46% of prescribers (Kidd et al., 2016). |  | Staff agree that guidelines are useful but there are discrepancies in how often staff use guidelines to guide their behaviour regarding testing and prescribing, which requires further exploration. |
| Attitudes towards Educational Interventions | Conflicting findings regarding need for education of nurses to influence prescribing (Gahr et al., 2007; Kid et al. 2016). | There was stronger support for integrating teaching about antimicrobial use during medical training (90%) and for antimicrobial stewardship training of medical coordinators (79%) (Kid et al., 2016). |  | Findings suggest that educational stewardship that addresses specific knowledge gaps can be helpful for nurses. However, educational stewardship alone is insufficient in shifting beliefs regarding awareness and psychosocial barriers. |
| Self-Confidence regarding Clinical Assessment and Prescribing: | Staff reported relatively high levels of confidence, however it was unclear if educational interventions improved confidence (Jump et al., 2015; Wilson et al., 2017; Hale et al., 2017; Drekonja et al. 2019) | Most prescribers felt positively regarding knowing when to order a urine culture, how to manage bacteriuria in a patient and being able to apply guidelines to patients. Despite these high confidence ratings, most prescribers reported lower scores for prescribing behaviours (e.g. prescribing based on type of infection even when there was no indication for prescribing) (Drekonja et al., 2019) |  | Findings suggest discrepancies between perception of confidence regarding assessment and prescribing for both, nurses and prescribers compared to guideline adherent behaviours. |
| AMR as a problem and Stewardship as an individual priority | All six studies found that RACF staff (nurses, prescribers) were aware of AMR (Wilson et al., 2017; Kid et al., 2016; Pringle et al., 2016; Gahr et al., 2007; Jump et al., 2015; Hale et al., 2017).  The beliefs among nurses about infection prevention being more important than antimicrobial stewardship and that nurses also reported that it can be hard to make antimicrobial stewardship a priority.  There were no significant changes in these beliefs after an educational intervention (p < 0.05) (Jump et al. 2015; Wilson et al., 2017). | All six studies found that RACF staff (nurses, prescribers) were aware of AMR (Wilson et al., 2017; Kid et al., 2016; Pringle et al., 2016; Gahr et al., 2007; Jump et al., 2015; Hale et al., 2017).  There were no significant changes in these beliefs after an educational intervention (p < 0.05) (Jump et al. 2015; Wilson et al., 2017). | All six studies found that associated healthcare staff (pharmacists and care managers) were aware of AMR (Wilson et al., 2017; Kid et al., 2016; Pringle et al., 2016; Gahr et al., 2007; Jump et al., 2015; Hale et al., 2017). | These findings suggest that there is a need to explore how staff, particularly nurses, feel about making stewardship a priority despite holding beliefs regarding the need for stewardship.  Current educational interventions aimed at targeting knowledge alone are not sufficient in changing nurses awareness or beliefs regarding stewardship. |
| Self-Efficacy/Perceived Behavioural Control | Nurses reported high confidence in their ability to contact prescribers to discuss infection symptoms and explain to resident/family why antibiotics are not necessary but these ratings did not change after the educational intervention (Hale et al., 2017).  A significant increase in nurses’ ability to tell if changes (to a patient’s clinical status) were due to an infection after the educational intervention (Wilson et al. 2017).  Nurses reported significantly higher change commitment and self-efficacy ratings compared with prescribers and nurses perceived medical providers’ readiness for change to be the same as their self-ratings (Scales et al., 2017). | Medical provider’s ratings of group readiness for change in nurses was lower than nurses’ ratings (Scales et al., 2017). |  | These findings indicate support that nurses feel positively about their ability to participate in stewardship, however it is unclear how other staff (e.g. prescribers, nursing assistants) feel about their ability to participate in stewardship |
| Perception of Role | Nurses perceive themselves to play a significant role in stewardship in being sources of information for patients regarding antibiotics, as well as their communication with providers (prescribers), knowledge of a patients’ baseline, clinical assessment of a patient and communication with patient and family members has influence on whether residents receive antibiotics (Ahouah et al. 2019; Wilson et al., 2017). | GPs perceive other aged-care staff, such as nurses, medical coordinators, and hospital specialists (ID or AMS team) to have a larger role in stewardship compared with themselves (Kid et al., 2016) | 72% of pharmacists endorsed the belief that they had a role to play in guiding antibiotic use in RACF’s but only 46% indicated that they reviewed medications and 36% provided training to aged-care staff, suggesting discrepancies between role perceptions and stewardship behaviours (Pringle et al. 2016). | There is agreement among nurses’ regarding their role in stewardship. However, it is unclear how often nurses feel they are able to fulfill this role. It is also unclear how nurses feel when they are unable to fulfil these duties as a result of organisational barriers (e.g. rotating prescribers, staffing shortages).  It is unclear how other RACF staff (e.g. prescribers, nursing assistants) perceive their roles in AMS.  Prescribers’ perceptions of their roles and responsibilities in stewardship needs to explored further. |
| Perception of Risk | No studies assessed the implications of professional risk for not prescribing antibiotics.  Mixed findings have been reported regarding nurses perceptions of risk to others. While the majority of nurses agree with statements that it is appropriate to wait in cases of uncertainty, 23% of nurses indicated need for antibiotics in an ASB vignette. | Limited studies have explored risk for prescribers. A study reported that prescribers had prescribed an antibiotic for the resident, only 40% felt it was “not at all likely” that the resident would have gotten better without the medication compared with 78% of nurses (Kistler et al., 2013). |  | These findings suggest that important considerations to staffs perception of risk have not been measured.  Further, there is a discrepancy between self-reported beliefs and behaviours that might lead to overprescribing of antibiotics and further attention needs to be paid towards addressing feelings regarding risk of serious repercussions to reducing prescribing (e.g. death, fear of litigation). |
| Social Norms and Team Culture | 33.1% of nurses did not perceive physicians to be committed to antibiotic stewardship and 28.3% of physicians were not perceived to have a good relationship with licensed nurses and 10.2% of nursing directors agreed that nursing staff ‘get no respect from physicians’ (Wagner et al., 2019).  Clinical Nurses Assistants (CNA’s) reported highest scores followed by staff providers (experienced prescribers), nurses, and then resident prescribers (Drekonja et al., 2019). | Gahr et al. (2007) found that although only 9.2% of physicians reported communication between nurses and physicians to be a problem, the majority of physicians also agreed that perceived pressure from nurses to order urine cultures contributed to antibiotic overprescribing. | Nurses are perceived to be the drivers of resident care by prescribers (Kid et al. 2016), social workers reported medical directors, directors of nursing and administrators rather than nurses to be perceived as the most influential in driving resident care (Lacey, 2005). | Findings suggest that there are inter-professional tensions that warrant further investigation. |
| Family Members and Resident Expectations | Nurses agree that antimicrobial requests from residents and their family members should not initiate calls to prescribers for antibiotics but nurses perceive that family members expect antibiotics when there is suspicion of an infection; and believe that antibiotics are associated with high quality care (Beeber et al., 2021). No changes in these perceptions were found after educational stewardship interventions (Wilson et al., 2017; Scales et al., 2017; Hale et al., 2017; Lacey, 2005; Ahouah et al., 2019). | Two studies compared nurses and prescribers’ perceptions and both studies found that significantly more nurses than prescribers endorsed the belief that antibiotics are expected and that family members influence prescribing decisions (Gahr et al., 2007; Scales et al., 2017).  Scales et al., (2017) reported that while both nurses and prescribers endorsed the belief that family members and residents’ had a preference for antibiotics, nurses’ rated the influence of family members and residents’ on prescribing significantly higher than prescribers. | In a study that asked social workers to identify the staff in facilities that have the most influence regarding medical interventions for residents, 20% responded “other”, with many specifying family members as most influential under this category (Lacey, 2005). | Findings suggest differences in the extent to which nurses and prescribers perceive resident and family members expectations regarding antibiotics to influence prescribing. It is unclear if this is reflective of closer proximity of nurses to residents and their families and warrants further exploration. |

Supplementary Table 5: PRISMA Search Strategy

| **Databases** | **Search terms** | **Limits and restrictions** | **Date of searches** |
| --- | --- | --- | --- |
| PsycINFO, PsycARTICLES, CINAHL Plus, MEDLINE, PubMed, Web of Science, Cochrane, and Scopus | (antimicrobial* stewardship* OR antibiotic* antibacterial* OR chemotherapy*) AND (agedcare*)  OR long-term aged-care* OR residential aged-care* OR homes for the aged* OR nursing homes* OR nursing facilities*) | Time period: 1990-2021 July; Language: English. | 31/07/2021 |
| PsycINFO, PsycARTICLES, CINAHL Plus, MEDLINE, PubMed, Web of Science, Cochrane, and Scopus | (antimicrobial* stewardship* OR antibiotic* antibacterial* OR chemotherapy*) AND (agedcare*)  OR long-term aged-care* OR residential aged-care* OR homes for the aged* OR nursing homes* OR nursing facilities*); | Time period: 1990-2019 August; Language: English. | 31/08/2019 |
